# NIpple Position to Pinpoint Localization of Chest Drain Insertion in FEmale Trauma Patients: The NIPPLE-Trial—A Landmark Study

**DOI:** 10.3390/jcm13216458

**Published:** 2024-10-28

**Authors:** Paula Beck, Mila M. Paul, Helena Düsing, Johanna C. Wagner, Sebastian Künle, Sebastian Imach

**Affiliations:** 1 Department of Orthopedics and Orthopedic Trauma Surgery, Schwarzwald-Baar Hospital Villingen-Schwenningen, 78052 Villingen-Schwenningen, Germany; sebastian.kuenle@sbk-vs.de; 2 Department of Orthopedic Trauma, Hand, Plastic and Reconstructive Surgery, University Hospital Würzburg, 97080 Würzburg, Germany; 3 Klinik für Unfall-, Hand und Wiederherstellungschirurgie, Universitätsklinikum Münster, Albert-Schweitzer-Campus 1, 48149 Münster, Germany; helena.duesing@ukmuenster.de; 4 Department of General, Viszeral, Transplantation, Vascular and Pediatric Surgery, University Hospital Würzburg, 97080 Würzburg, Germany; wagner_j6@ukw.de; 5 Department of Trauma and Orthopedic Surgery, Cologne Merheim Medical Center (CMMC), University Witten/Herdecke, 51109 Cologne, Germany; imachse@kliniken-koeln.de

**Keywords:** chest drain, polytrauma, nipple, thoracic trauma, gender bias

## Abstract

**Background**: The insertion of chest drains (CD) in trauma patients is a lifesaving, albeit high-risk intervention. Safe insertion of CD in settings where aids like ultrasound are not available relies on a landmark technique defining the so-called triangle of safety. The inferior margin of this triangle is identified by nipple height, which is thought to correspond to the fifth intercostal space (ICS). Training manikins are modeled after a lean male body and oftentimes insinuating jokes fuel uncertainty about the height of the nipple as a reliable landmark in female trauma patients. This study aims to prove that the nipple can be considered a safe landmark for CD insertion in women: even if larger breasts follow the force of gravity, it should not act towards the direction of the feet, but to the sides of the thorax in a flat-lying trauma patient. **Methods**: An online questionnaire was designed and distributed amongst female surgeons. Epidemiological data (age, nr of pregnancies, history of breastfeeding) was collected. Height, weight, and body mass index (BMI) were reported. Chest circumference and underbust girth were self-measured. The nipple position in relation to the ICS was measured by the women while lying in a supine position. **Results**: 237 female surgeons completed the questionnaire. Nine questionnaires were excluded due to incomplete data sets. 20 women were excluded due to previous breast surgery. Thus, 208 participants (or 416 nipples) were included in analysis. In supine position, both nipples were located in the 5th (±0.8) intercostal space. Age, BMI, height, weight, and status post-pregnancy had no significant impact on nipple position. Interestingly, a history of breastfeeding correlated with lower nipple position. **Conclusions**: The nipple is a safe landmark for identifying the correct insertion position of chest drains in supine-lying female trauma patients.

## 1. Introduction

50% of all polytraumatized patients sustain a relevant chest injury, and thoracic injuries account for 25% of trauma-related deaths worldwide [1,2]. Severe thoracic trauma has a high mortality rate of around 30%, which emphasizes the need for fast diagnosis and treatment [2].

A common, immediate treatment option for thoracic trauma, such as tension pneumothorax or hemothorax, is the placement of a chest drain (CD) [3]. In Germany, around 10% of severely injured patients receive a CD in the emergency department during or after the primary survey [4].

The most common error while placing a CD is the incorrect identification of the triangle of safety [5]. A recent systematic review and meta-analysis showed an overall complication rate of 19% in CD insertion, and over half (53.1%) were due to positional mistakes [6]. Thus, simplifying the correct tube placement is extremely important. In settings where aids like ultrasound are not available, landmark methods are used to select the correct entry point for CD insertion:

The recommended incision for finger thoracostomy and CD is the so-called “triangle of safety” below the axilla. The triangle is defined by the margin of the musculus latissimus dorsi posteriorly and the margin of the musculus pectoralis major anteriorly Its base is defined by a transverse line through the nipple which is thought to correspond to the 5th intercostal space (ICS) [7]. Although the established trauma courses (ATLS, ETC) teach this method, reliable trials confirming the nipple as a landmark for the 5th ICS are scarce, and recent cadaveric and ultrasound-guided studies challenge the accuracy of this approach, especially in female patients [8,9].

In teaching situations, it is often said (although this statement is rarely found in writing) that the nipple as a landmark is only valid for male patients, and can be significantly lower in women, depending on their constitution [4]. Depending on breast size and the condition of the connective tissue, a female breast could sag and the nipple could therefore be located inferior to the 5th ICS.

Additionally, manikins used for teaching the correct insertion of a CD resemble the shape of a lean, “standard male” thorax. This may lead to insecurities while treating female trauma patients and even result in a less favorable outcome.

Such a gender bias has already been shown for other trauma interventions, for example time to triage, pelvic binder application, or tranexamic acid administration [10,11].

However, as CD placement in trauma is usually done with patients positioned supine, if gravity influences the position of the nipple in individuals with bigger breasts, it should act sideways along the thorax in the direction of the stretcher and not in the direction of the feet. Thus, we hypothesize that the transverse nipple line is a reliable landmark for the fifth ICS regardless of age, body mass index, and history of pregnancy and breast feeding in supine lying women.

## 2. Methods

An online questionnaire (see Supplementary Material) in German language was designed utilizing the Evasys V9.1 online questionnaire tool licensed for Würzburg University. The link to the questionnaire was then distributed amongst the German Women In Surgery network (“die Chirurginnen e.V.”).

Further distribution among surgical peers was encouraged.

The questionnaire was anonymous. It was accessible online for one month.

Female surgeons were eligible to participate in the study. Epidemiological data (age, number of pregnancies, history of breastfeeding) was collected. Height and weight were reported, and body mass index (BMI) was calculated. Chest circumference and underbust girth were self-measured, and the results were reported.

The measurement of the nipple position was performed via self-assessment by the women: While lying in a supine position, the sternoclavicular joint was identified via palpation. From there, individual intercostal spaces were palpated and counted from cranial to caudal until the level where the nipple was located. This maneuver was then repeated for the other breast. A detailed instruction of how to identify the individual intercostal spaces was provided in the questionnaire form.

Statistical analyses were performed with Sigma Plot (Version 13, Systat Software). Shapiro-Wilk was used to test normality. If data were not normally distributed, we used the non-parametric Mann-Whitney rank sum test for statistical analysis and reported data as median (25th–75th percentile). Asterisk indicates significance level (* *p* < 0.05) and *n* denotes the sample number. Linear regression curves were fitted and Pearson correlation coefficient r and statistical significance *p* of correlations were evaluated in Sigma Plot. All plots were produced with Sigma Plot. Figures were assembled using Adobe Illustrator (28.6 © 1987-2024 Adobe).

The present study was approved by the ethics committee of the Faculty of Medicine, University of Witten/Herdecke, Germany (No. S-205/2024). Patients or the public were not involved in the design of this trial.

## 3. Results

A total of 237 women returned a questionnaire. Nine questionnaires were excluded due to incomplete data sets. 20 women were excluded due to previous breast surgery. Thus, 208 participants (416 nipples) were included in the analyses (Table 1).

**Figure 1:**
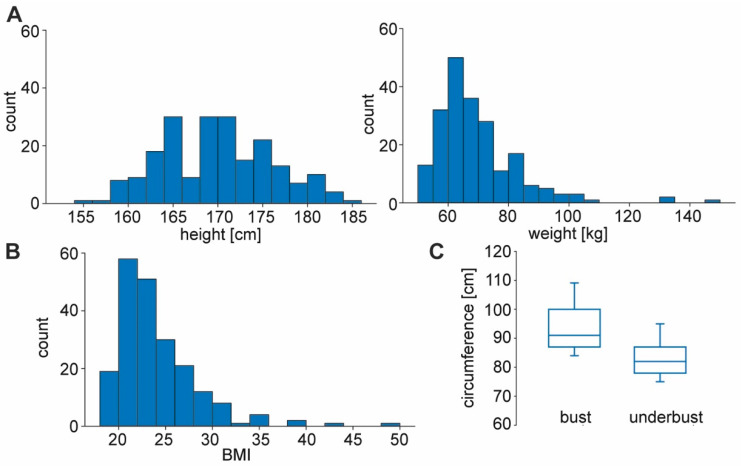
Body dimensions of the study group. (**A**) Histogram of height (in cm) and weight (in kg) of all 208 participants. Median height was 169 cm (± 6.1 SD) with 184 cm maximum and 155 minimum. Median weight was 65 kg (± 13.7 SD) with 149 kg maximum and 50 kg minimum. (**B**) Histogram of BMI in the participants (22.9 ± 4.3 median ± SD). (**C**) Participants were asked to measure their bust circumference (at the nipple level) and underbust circumference (directly below the breasts). Box plots show bust and underbust circumferences (91 ± 10.7 cm median ± SD; 82 ± 8.7 SD). Boxes represent median, 25th and 75th percentiles, whiskers 10th and 90th percentiles.

Mean height was 169 ± 6 cm, and mean weight was 65 ± 14 kg. The calculated mean BMI was 22.9 ± 4.3 kg/m^2^. Mean chest circumference was 91 ± 10.7 cm, and mean underbust girth was 82 ± 8.7 cm (Figure 1).

**Figure 2:**
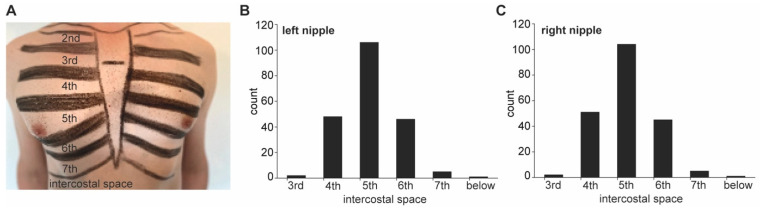
Measurement technique for estimation of nipple level assigned to the intercostal space. (**A**) Measurement technique for localization of nipple position in a male anterior thorax. (**B**,**C**) Histograms of the respective intercostal spaces to which left (**B**) and right (**C**) nipples could be allocated to. More than 50% of the nipples were assigned to the 5th intercostal space (5th ± 0.8 SD for left and right, respectively). However, in 11.8% of the women, left and right nipples were assigned to different intercostal spaces.

In the supine position, both nipples were located in the 5th (±0.8) intercostal space. In 11.8% of the women, left and right nipples were located in different intercostal spaces (Figure 2). 

**Figure 3:**
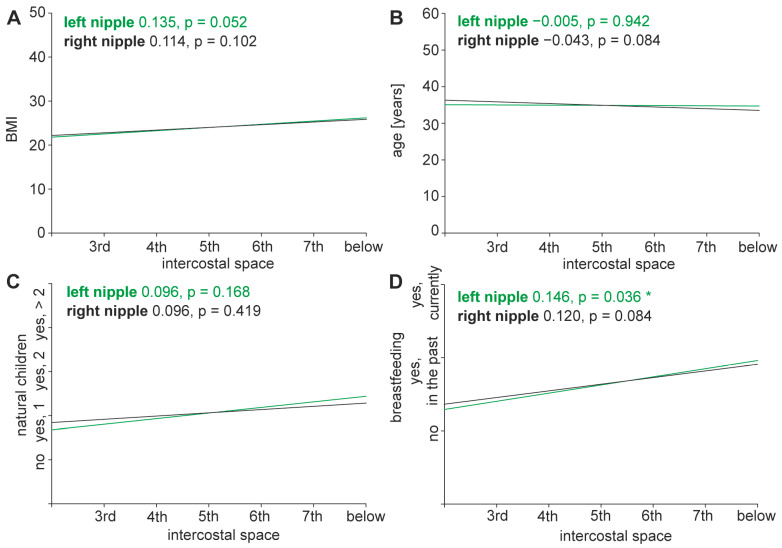
Correlation of nipple height with body dimensions, age, natural children and breastfeeding. (**A**–**D**) Correlations between nipple height assigned to different intercostal spaces and possible external influences were calculated using Pearson correlation coefficients. Height of left and right nipples versus BMI (**A**), age (**B**), natural children (**C**) and breastfeeding (**D**) are shown. Positive correlation coefficients with *p*-values below 0.05 increase together, negative correlation coefficients with *p*-values below 0.05 correlate opposed, pairs with *p*-values greater than 0.05 show no significant correlation. Asterisk (*) indicates *p*-value < 0.05.

Age, BMI, height, weight, and status post-pregnancy had no significant impact on nipple position. A history of breast-feeding correlated with a lower nipple position but did not reach statistical significance (Figure 3).

## 4. Discussion

This study validated the female nipple as a reliable landmark for locating the 5th ICS in supine lying women.

On average, the 208 test subjects located their nipple at the level of the 5th ± 0.8 ICS. In almost 12% of the women, left and right nipples were located on different ICS.

BMI (*p* = 0.052 for left nipple, *p* = 0.102 for right nipple), age (*p* = 0.942 for left nipple, *p* = 0.541 for right nipple), and previous pregnancy (*p* = 0.168 for left nipple, *p* = 0.419 for right nipple), had no statistically significant effect on the height of the nipple.

A history of breastfeeding correlated with lower nipple position and reached statistical significance for the left nipple (*p* = 0.036 for left nipple, *p* = 0.084 for right nipple), but this statistical finding does not reach clinical significance.

The main hypothesis can therefore be verified: The nipple is a reliable landmark for the 5th ICS in supine-lying women. Chest drains inserted above the nipple in the anterior axillary line are located in the 4th or 5th ICS and should therefore be safely above the level of the diaphragm.

In recent years, it has become apparent that women do not receive the same standard of care as men do after being critically injured. This includes, for example, the administration of tranexamic acid or the application of a pelvic binder, but also the time until triage [10,11].

There are time-critical interventions in the care of seriously injured patients. One of these is the placement of a CD, e.g., in the case of tension pneumothorax [3].

The insertion of CD in trauma patients is a lifesaving but high-risk intervention and requires extensive training and supervised learning.

The manikins utilized for teaching CD insertion are thoraces without breasts—corresponding to a lean, “standard male” thorax. Furthermore, the current course formats (ETC, ATLS) often teach the height of the nipple as a landmark for the correct placement of a CD [7]. Such training geared towards male anatomy, together with the uncertainty caused by insinuating jokes about nipple height in women, could lead to CD being placed less safely and perhaps less consistently in women than in men.

To date, landmark studies involving nipple position have been mainly done with formalin-fixated body donors [9]. The authors are not aware of any literature validating the nipple as a landmark with a substantial cohort of alive test subjects. Studies with small numbers of participants show results that differ from cadaver studies [8]. However, the widespread doctrinal statement considering the nipple as a landmark for the 5th ICS is based primarily on hearsay and decade-old data [12].

This study, on the other hand, shows that the nipple can serve as a reliable landmark for the correct placement of a chest tube in supine lying women.

Some limitations need to be discussed:

Mean age of the participating women does not represent the average German population as the test subjects were recruited mainly from the Chirurginnen-Network and are therefore younger than the average population. However, age had no significant impact on the nipple position in supine position in our cohort. But, as there is a lack of data, recommendations for the geriatric population need to be given with caution.

Mean BMI of the participating women was 22.9 ± 4.3 kg/m^2^, which is below mean BMI for women in Germany. Further studies will be needed to determine if the results apply to obese individuals, regardless of gender.

In the questionnaire, ethnicity was not queried. Thus, no statement can be made in this regard.

The participants were responsible for determining the location of their nipples in relation to their ICS, which could not be controlled by the authors. Detailed instructions were provided, nevertheless, measurement errors or uncertainties regarding the own anatomy and the correct numbering of the ICS could have occurred. However, the participants were all doctors, most of them surgeons. Surgeons routinely perform incisions based on palpation of anatomical landmarks. A sound anatomical understanding can therefore be assumed, and the data collected can be considered to be reliable. However, a measurement error cannot be ruled out with absolute certainty.

Measurement was performed with the arm lying on the side of the thorax. When inserting a CD to treat an intrathoracic pathology, the ipsilateral arm is elevated behind the back of the head. The nipple might then move. This was not accounted for in the present study design, but if there is a change of nipple position the nipple would be found more cranially if the ipsilateral arm is elevated [13]. This makes the nipple as a landmark even safer.

In conclusion, the main finding is not compromised by the aforementioned limitations: The NIPPLE-Trial suggests that the nipple could be considered a safe landmark to identify the correct insertion position of CD in supine lying female trauma patients. It therefore makes an important contribution to closing the gender knowledge gap in emergency medicine. A simple rule of thumb applies to everyone, not just men: The nipple can be used to determine the height of the correct ICS when performing a thoracostomy. Patient safety is increased.

Thus, this study does not only close a gender data gap, it creates a new one: to date, there is no solid scientific proof for the claim that nipple position can serve as a reliable landmark for safe chest drain insertion in male patients.

Further studies are needed.

## Figures and Tables

**Table 1:** Characteristics of the study population. This table shows age distribution, body measurements, the number of natural children and previous breastfeeding in the study population. Of all 237 participants 20 underwent breast surgery in the past and were excluded from further analysis, 9 participants were excluded due to inconclusive results during measurements. Thus, 208 participants were included into analyses. The respective values for all parameters are given in absolute terms and in percent of the study group (*n* = 208 women).

Characteristics (*n* = 237)	*n*	*n* (in %)
Previous breast surgeries		
yes	20	8.4
no	217	90.7
Characteristics (*n* = 208)	*n*	*n* (in %)
Age [years]		
21–30	29	13.9
31–40	95	45.6
41–50	59	28.3
>50	25	12
Body measurements [mean]		
height [cm]	169	-
weight [kg]	65	-
BMI	22.9	-
Natural children		
yes, one	33	15.8
yes, more than one	85	40.8
no	90	43.2
Previous breastfeeding		
yes, currently	14	6.7
yes, in the past	103	49.4
no	91	43.7

## Data Availability

The original contributions presented in the study are included in the article/Supplementary Material, further inquiries can be directed to the corresponding author/s.

## Supplementary Materials

The following supporting information can be downloaded at: https://www.mdpi.com/article/10.3390/jcm13216458/s1, NIPPLE original questionnaire.
