# Supplementary material for: NIpple Position to Pinpoint Localization of Chest Drain Insertion in FEmale Trauma Patients: The NIPPLE-Trial—A Landmark Study
Source: J Clin Med. 2024 Oct 28;13(21):6458. doi: 10.3390/jcm13216458 (PMC11546316; doi:10.3390/jcm13216458)
Supplement: Supplementary file 1 [file jcm-13-06458-s001.zip › jcm-3245036-supplementary.pdf]

MUSTER

Bitte so markieren: ☐ ☒ ☐ ☐ ☐ Bitte verwenden Sie einen Kugelschreiber oder nicht zu starken Filzstift. Dieser Fragebogen wird maschinell erfasst.
Korrektur: ☐ ■ ☐ ☒ ☐ Bitte beachten Sie im Interesse einer optimalen Datenerfassung die links gegebenen Hinweise beim Ausfüllen.

1. Persönliche Angaben und subjektive Messwerte

Schwerverletzte Patientinnen werden flach auf dem Rücken liegend versorgt. Die Mamille wird in diesem Fall unabhängig von Brustgröße und -form sowie allgemeiner Konstitution auf Höhe des 5. Interkostalraumes zu finden sein. Somit ist die Mamille bei flach liegenden Frauen eine sichere Landmarke zum Auffinden der korrekten Höhe bei Anlage einer Thoraxdrainage. Diese Hypothese möchten wir im Rahmen des aktuell vorliegenden Fragebogens mit Ihrer Hilfe überprüfen. Die Befragung ist freiwillig und vollständig anonym. Sie können die Bearbeitung des Fragebogens jederzeit ohne Angabe von Gründen abbrechen.
Wir bedanken uns im Voraus herzlich für Ihre Unterstützung!

1.1 Ich habe die Aufklärung zur Teilnahme am NIPPLE-Trial gelesen, habe keine Fragen und bin mit der anonymen Teilnahme an der Studie einverstanden.
☐ ja ☐ nein

1.2 Bitte nennen Sie Ihr Alter.
☐ 21-30 ☐ 31-40 ☐ 41-50
☐ 51-60 ☐ 61-70 ☐ 71-80

1.3 Wie groß sind Sie (in cm)?

1.4 Wie schwer sind Sie (in kg)?

1.5 Wurden Sie jemals an einer oder an beiden Mammae operiert?
☐ ja ☐ nein

1.6 Haben Sie leibliche Kinder?
☐ nein ☐ ja, eins ☐ ja, zwei ☐ ja, mehr als zwei

1.7 Haben Sie in der Vergangenheit eins oder mehrere Ihrer Kinder gestillt oder stillen aktuell?
☐ nein ☐ ja, in der Vergangenheit ☐ ja, ich stille aktuell

1.8 Wie groß ist Ihr Brustumfang auf Mamillenhöhe in liegender Position (in cm)?

1. Persönliche Angaben und subjektive Messwerte [Fortsetzung]

1.9 Wie groß ist Ihr Unterbrustumfang (gemessen auf Höhe der Mammaumschlagsfalte) in cm?

1.10 Auf welcher Höhe befindet sich in flach liegender Position die Mamille Ihrer LINKEN Brust? (Bitte tasten Sie hierzu zunächst das Sternoclaviculagelenk. Unmittelbar kaudal davon befindet sich der 1. Interkostalraum (ICR). Bitte zählen Sie nun die einzelnen ICR von kranial nach kaudal ab, bis Sie schließlich den erreichen, auf dessen Höhe sich in flacher Rückenlage die Mamille Ihrer Brust befindet.)

- ☐ 3. ICR
- ☐ 4. ICR
- ☐ 5. ICR
- ☐ 6. ICR
- ☐ 7. ICR
- ☐ unterhalb des 7. ICR

1.11 Auf welcher Höhe befindet sich in flach liegender Position die Mamille Ihrer RECHTEN Brust? ((Bitte tasten Sie hierzu zunächst das Sternoclaviculagelenk. Unmittelbar kaudal davon befindet sich der 1. Interkostalraum (ICR). Bitte zählen Sie nun die einzelnen ICR von kranial nach kaudal ab, bis Sie schließlich den erreichen, auf dessen Höhe sich in flacher Rückenlage die Mamille Ihrer Brust befindet.)

- ☐ 3. ICR
- ☐ 4. ICR
- ☐ 5. ICR
- ☐ 6. ICR
- ☐ 7. ICR
- ☐ unterhalb des 7. ICR
